# Supplementary material for: Complexome profiling of the Chlamydomonas psb28 mutant reveals TEF5 as an early PSII assembly factor
Source: Plant Cell. 2025 Mar 17;37(6):koaf055. doi: 10.1093/plcell/koaf055 (PMC12164590; doi:10.1093/plcell/koaf055)
Supplement: koaf055_Supplementary_Data [file koaf055_supplementary_data.zip › psb28_interactive.zip!/plots/intensity/qualityPlots/peptides/FAD6_Cre13.g590500.t1.1.html]

### Cre13.g590500.t1.1

Trivial name: FAD6  
SeqCoverage [median]: 5.20  
Mass: 48.38 kDa  
  
 

| Sequence | MissedCleavage | Mass [Da] | wtIntensity | muIntensity | Score |
| --- | --- | --- | --- | --- | --- |
| EEALFAVQR | 0 | 1061.55 | 656.41 | 0.00 | 100.19 |
| ELPDNVSLTDIIK | 0 | 1455.78 | 2789.82 | 0.00 | 83.86 |
|  |  |  | 3446.2 | 0.0 |  |
